# Back to the future: cordycipitoid fungi in a postgenomic world

**DOI:** 10.1080/21501203.2017.1385545

**Published:** 2017-11-22

**Authors:** Richard A. Humber

**Affiliations:** USDA-ARS Emerging Pests and Pathogens Research, Robert W. Holley Center for Agriculture and Health, Ithaca, NY, USA

**Keywords:** Nomenclature, 1F = 1N, typification of scientific names, genomic databases, fungal endophytes, rhizosphere associations, pharmacology, biochemistry, developmental biology

## Abstract

The massive – but nearly uninterpretable – work of Kobayasi on *Cordyceps* species (many of which were illustrated gorgeously by D. Shimizu’s paintings) was made useful and modernised by the phylogenetically based reclassification by Gi-Ho Sung and others in 2007. The rejection of dual nomenclature in favour of the new standard for the names of pleomorphic fungi that allow only a single valid name for any one organism has resulted in uncertainties and confusions about what names should be used, and these doubts will certainly continue until at least the 2023 International Botanical Congress. Enough genomic and molecular information has now accumulated about so many of these cordycipitoid fungi, however, that it is appropriate to step back from the thermocyclers that now dominate so many biological laboratories to consider how these genomic findings may be used to advance our understanding of these fungi and their activities in the real world (as opposed to their genomic dissections in a thermocycler). Questions to be considered include how genomic information might help guide future research on such diverse issues as understanding the natural ecology of these fungi, the interactions of biotic and abiotic factors needed to initiate their sexual stages, and better clues about how to elicit the productions of a wide range of biologically active compounds known to be produced by cordycipitoid fungi.

The Cordyceps Forum 2016 conference placed a strong focus on the ongoing exploration of the genomics of *Cordyceps* and its innumerable hypocrealean relatives, both teleomorphic and anamorphic as well as on the pharmacological capabilities of these fungi. This presentation, however, was planned to examine a very different perspective on these fungi by trying to look backwards into more traditional (and pre-genomic) perspectives on mycology in order to look forward to what might actually be done with the incredible profusion of genomic data and, indeed, full organismal sequences that are now accumulating.

Before doing that, however, it is worth noting that there has been significant progress in uncovering a significant amount of new biodiversity of *Cordyceps* and related cordycipitoid taxa in such underexplored parts of the world as the Amazonian River Basin in Colombia and Brazil and that two young and extremely active scientists need to be singled out for their continuing contributions on these fungi in that part of the world: Tatiana Sanjuan, who spoke at the CordyForum 2016, has concentrated on Colombian collections (Sanjuan et al. 2001, 2014, 2015) but has been active in other parts of the world as well. Especially intriguing among Sanjuan’s findings is the description of more teleomorphic states for fungi whose anamorphs are referable to *Beauveria* (Sanjuan et al. 2014). João Araújo, who is completing PhD studies at the Pennsylvania State University in the US, has concentrated on the *Ophiocordyceps* species affecting ants in the Brazilian Amazon (Araújo et al. 2015; Araújo & Hughes 2016) and has a major manuscript in a late state of preparation (intended for publication in Studies in Mycology) describing a large number of new species, most of which are in the *Ophiocordyceps unilateralis* species complex; he not only is a well-skilled mycologist, but also brings great artistic accomplishment via photography, drawing, and painting skills to document his findings.

In a symposium on the genomics of entomopathogenic fungi at the 2005 meeting of the Society for Invertebrate Pathology in Anchorage, Alaska, a number of interesting points were raised that need to be recalled now: At that time, the sequencing of full genomes of *Beauveria bassiana* and *Metarhizium anisopliae* was keenly anticipated but had not yet occurred. Neither, of course, had the phylogenetically based reclassifications of species in these two genera (Bischoff et al. 2009; Rehner et al. 2011; Kepler et al. 2014) yet happened, with their corresponding fragmentations of the well-recognised but morphologically defined species into multiple phylogenetically defined ones … and automatically placing some quotation marks around the base taxa in what we now know retrospectively to be major species complexes. During that symposium, I expressed hope that the next entomopathogenic fungus to be sequenced after these two critically important taxa would be *Cordyceps militaris*. My reason for this preference was that this easily cultured and easily fruited teleomorph could and should serve as some sort of Rosetta Stone to open and to illuminate many diverse lines of experimental and ecological approaches to understanding all aspects of the biology of these and all other cordycipitoid teleomorphs. An examination of some of those activities that could be enabled by a full sequence of *C. militaris* forms a major focus of this presentation.

Basith and Madelin (1968) reviewed the earliest history of obtaining fruitings of *Cordyceps* isolates in cultures. It has been indisputable that *C*. *militaris* has been the easiest and most reliable species for inducing production of the sexual stromata *in vitro*, and this species is now being commercially produced in many facilities throughout East Asia. Much more recently, there has been significant progress towards obtaining *in vitro* fruitings of *Ophiocordyceps sinensis* (Cao et al. 2015; also see this Cordyceps Forum’s presentation by Taihui Li); this advance is enormously important because such fruitings are apparently being accomplished in vitro on rice-based medium the absence of the natural hepialid insect larval host of this fungus, and at a much lower altitude than the normal habitat for this fungus on high in the Himalayan Mountains and Tibetan Plateau. Induced insect-free *in vitro* fruitings of *O. sinensis* may prove to be something of a mixed blessing: the strong possible commercial and scientific benefits of this advance are indisputable and provide hope that some of the excessive environmental pressures on both the fungus and its hosts due to the reported overharvesting of the naturally occurring populations of both the fungus and its host might be partially relieved (Qiu 2013; Shrestha and Bawa 2013), but removing some of the dependence on naturally occurring collections of *O. sinensis* might also seriously threaten the life styles and economic well-being of those living in the natural ranges of this fungus who depend on the annual harvest and sale of *O. sinensis* fruiting bodies.

## New nomenclatural rules affect all *Cordyceps* and related hypocrealean species

Much progress on cordycipitoid fungi has been made in recent years in understanding their taxonomy, systematics, and biodiversity. These areas of mycological activity will certainly continue to expand rapidly, and many more of these fungi will be described despite the current difficult transition from the old dual nomenclatural rules in effect before 2012 that allowed separate, valid names for the anamorphs and teleomorphs of these fungi to the new rule (in effect since 1 January 2012) permitting only one name for any one organism (for mycologists, this new rule is widely known as One Fungus = One Name, or 1F = 1N). This transitional period is made more difficult because the knowledge about cordycipitoid fungi is expanding so quickly while the number of genera and species among which they may be classified is simultaneously being reduced (and still unsettled at the time of Cordyceps Forum 2016). The new nomenclatural rule is actively forcing the creation of mycological “black holes” that were foreseen by Humber and Rombach (1987) as a result of amalgamating sometimes extremely dissimilar anamorphic and teleomorphic genera into revised, expanded genera that will no longer be based on the sorts of predictive and informative generic concepts that have been so useful under the now prohibited dual nomenclatural system.

While most biologists may be deeply disturbed by the loss of meaningful, predictive generic concepts for so many fungi, it is important to note that the Preamble of the International Code of Nomenclature for algae, fungi and plants (ICN; McNeill et al. 2012) includes an ominous sentence whose meaning has now become chillingly apparent under the 1F = 1N rule (with emphasis added): “The purpose of giving a name to a taxonomic group is *not to indicate its characters or history, but to supply a means of referring to it and to indicate its taxonomic rank*.”

Precisely how the new nomenclatural standard will shape the systematics and taxonomy of hypocrealean (and so many other!) fungi is still a work in progress that cannot be completed before the closing of the 19th International Botanical Congress (IBC) in Shenzhen, China, at the earliest or, in a worst case scenario, not until the end of the 20th IBC in 2023 at a still undetermined location. The mechanics of how decisions are being made to choose the names of genera allowed to remain in use under the new rules and the many to be rejected are complex and involve consortia of experts in individual fungal groups, then a series of committee decisions within the International Association for Plant Taxonomy that will culminate in a vote at the end of the week-long Nomenclature Section of the Shenzhen IBC, and finally ratified by a general vote held during the final plenary session of the IBC (see Humber 2016).

The venerable and long-used taxonomy of *Cordyceps* in its broad sense is derived from the monumental, life-long efforts of Kobayasi (1941, 1982) and in innumerable other separately published papers as well as in books with supremely important watercolour illustrations of *Cordyceps* species (Kobayasi and Shimizu 1983; Shimizu 1994). The photographic atlas of Korean collections of *Cordyceps* and related entomopathogenic fungi (Sung 1996) has provided an important photographic supplement to Shimizu’s watercolours and other illustrations of many of the same cordycipitoid fungi. The major achievement bringing the Kobayasi classification up to the modern, phylogenetically based systematics standards led Sung et al. (2007) to a massive reclassification of the great majority of the older names in *Cordyceps* among a series of genera in a set of three families – Clavicipitaceae, Cordycipitaceae, and Ophiocordycipitaceae – in the order Hypocreales. The phylogenetic revisions of the anamorphs of these cordycipitoid fungi began with the phylogenetic reclassifications of *Verticillium* section *Prostrata* as originally recognised by Gams (1971) into a series of phylogenetically based segregate genera (Zare et al. 2000, 2001a; Gams and Zare 2001; Sung et al. 2001; Zare and Gams 2001). Soon after the reworking of entomopathogenic *Verticillium* species, Samson’s (1974) classic taxonomy that recognised *Paecilomyces* section *Isarioidea* was also revised phylogenetically (Luangsa-ard et al. 2004), with most species assigned to the genus *Isaria* (Luangsa-ard et al. 2005) but also with another important species, *P. lilacinus*, later reclassified into the ophiocorydcipitaceous genus *Purpureocillium* (Luangsa-ard et al. 2011). It appears that the new classifications under the 1F = 1N standard are now more or less in place for the entomopathogens in the Clavicipitaceae (Kepler et al. 2014) and Ophiocordycipitaceae (Quandt et al. 2014) that include some surprising amalgamations of genera; no new classification of the Cordycipitaceae has yet been published (partly because of continuing uncertainties about the final list of genera and subgenera to be accepted).

## Importance of using type specimens and ex-type isolates

No revisions of taxonomy can be undertaken without reference to the initially published concepts on which every described species must be based to the appropriate type specimens and/or any cultures derived from or used to generate the permanently deposited type specimens of their taxa. The ICN defines several classes of nomenclatural types that defined in large part by whom (and when) any type was designated. More recently, the issue of epitypification has become prominent (Hyde and Zhang 2008) as a vitally important way to provide access to information (molecular or otherwise) that is unobtainable from a permanently preserved holotype, lectotype, or neotype specimen. Permanently preserved epitype specimens are intended to provide a useful example of how a given species name should be understood and interpreted into the future; epitypes have a high nomenclatural status and must be chosen wisely and with great care. The greatest importance of epitypifying many fungi, however, may be that the designated specimens are either prepared from a living culture or the epitype specimen was the source for a living culture; these ex-epitype cultures then become available, hopefully in perpetuity, for any and all ecological, physiological, molecular, developmental, systematic, genetic, genomic, or any other type of studies needing to include such critically important, taxonomically verified reference materials.

The sequence data generated from isolates may be completely correct and reproducible but may also have little if any inherent value if the source organism was incorrectly identified or its identity was in any way questionable. Further, the use of such data from organisms whose names are not recorded correctly in any and all later studies can be completely misleading or, in some instances, even scientifically dangerous (Morrison et al. 2009; Prie et al. 2012). Identifications of an organism and comparisons among groups of organisms can be truly meaningful and reliable only when there are one or more organisms in the study whose identity is beyond any possible doubt.

## Using genomic databases: are the data trustworthy or not?

Genomically based taxonomic approaches are now functionally obligatory throughout the mycological world. This circumstance is due to the inherent power of sequence comparisons to confirm the identification of some known taxa or to provide convincing reasons to believe that an unidentified organism being compared with others may be new or at least different from what has been studied to date. This last condition – being verifiably distinct from the sequence data accumulated for similar taxa in the genomic databases such as GenBank (http://www.ncbi.nlm.nih.gov/genbank) – may be one of the major shortcomings in the way modern systematics is now operating. Traditional taxonomic approaches inherently understood that the identifications of fungi (indeed, all organisms) could be trusted only after comparisons with the established, permanently preserved type specimen and/or against organisms that may have been verified to match the characteristics of the type specimen that was identified at the time of original publication of a name (or through any of a series of other possible circumstances that might involve the various classes of types that are recognised in the ICN).

Authentic and type collections (whether they are specimens, cultures, or DNA sequences derived from such materials) can be accepted without question as correctly identified and useful standards on which to base taxonomic comparisons. There is a much lower reliability when any organismal identification has not been explicitly justified or supported in a publication noted in the background data submitted to a genomic database together with the sequences or if (in too many instances) the initial identification of the DNA’s source organism was a matter of guesswork rather than accurate taxonomic verification. It is possible that organisms from which cited sequence data were obtainedmay be correctly identified, even if by guesswork (similarity to published illustrations, whether or not the illustrations were of the nomenclatural type, or by a substantial if imperfect matching of the physical characteristics of an organism to what has been published elsewhere), that correctness really needs to be verified if any weight is to be placed on an identification that is not based on some sort of type or ex-type material. When an organism has been misidentified, whether by accident or because the organism’s taxonomy was revised (with a resultant segregation of new, more narrowly circumscribed taxa segregated from broadly described ones), it is important to be aware that the records about the original identification of an organism as submitted to genomic databases are rarely or ever changed post facto to reflect any later determined identification of the source organism. These complications obviously damage or destroy the usefulness of any analyses incorporating sequences from such incorrectly identified organisms.

If one seeks to use GenBank’s vast genomic resources and BLAST searches (http://blast.ncbi.nlm.nih.gov/Blast.cgi) to identify an organism from which one has obtained new sequences, the search output lists the matching sequence accession numbers in decreasing degree of similarity with one’s test sequence. Even if the search reveals a 100% sequence congruity between the unknown and a sequence retrieved by that BLAST search, this cannot necessarily be taken as proof of identity (as discussed in the previous paragraph). If there is a complete or close match against only one gene sequence, there is no guarantee that one’s unknown test sequence and its match from the database might not differ significantly when considering some other genes accepted to have taxonomic value. ITS (Internal Transcribed Spacer) sequences have been widely accepted and promoted as a supposedly universal DNA “barcode” and, indeed, have adequately differentiated a huge variety of fungi. Nonetheless, it must be noted that for the Hypocreales – and especially for cordycipitoid entomopathogenic taxa – ITS has proven to have little to no taxonomic value (Driver et al. 2000; Bischoff et al. 2009). ITS sequences were excluded altogether from the analyses providing the new phylogenetic reclassifications of the genera *Beauveria* and *Metarhizium* as well as of the families Clavicipitaceae and Ophiocordycipitaceae (Rehner et al. 2011; Kepler et al. 2014; Quandt et al. 2014).

Some additional issues about the attributions of the sources of materials being sequenced and included in genomic databases directly affect the trustworthiness and utility of the data: When trying to place genomic studies into the broader biological concept, it is important to know how reliable the identification may be for the source of the sequences, but it may also be important to understand the history and provenance of a given fungal isolate because genomic databases do not require that vouchers of the DNA source organisms be deposited in established culture collections. The auxiliary data that depositors provide with their sequences usually provide very little information about the source or (cultural) history of the organism. The need to reproduce or to verify data of these cultures is a basic requirement for respectable science, but this need is violated if the source organisms are unavailable or untraceable. It is very helpful when sequence depositions are based on isolates that have come from general service culture collections such as (for entomopathogenic fungi) the USDA-ARS Collection of Entomopathogenic Fungal Cultures (ARSEF; Ithaca, NY, USA), CBS (Utrecht, The Netherlands), the CABI Genetic Resource Centre (IMI; Egham, UK), German Collection of Microorganism and Cell Cultures (DSMZ; Darmstaadt, Germany), and American Type Culture Collection (ATCC; Manassas, VA, USA). There are innumerable instances in the literature where blocks of cultures have been received from one or more of these culture collections and then relabelled with a local collection designation that, sadly and inappropriately, may be used in publications rather than the original culture collection identifiers and accession numbers. Such a practice is damaging for the culture collections from which the cultures were obtained and also prevents appropriate understandings about later published studies that might have used the same isolates without indicating the original sources. This, in turn, greatly complicates or inhibits the ability of other scientists to obtain, to work with, or to evaluate the same isolates for their own research needs.

## Alternative lives for some of a few cordycipitoid entomopathogens

Even only a relatively few years ago, there was a broad recognition and expectation that fungal pathogens of insects were treated as entomopathogenic fungi that, in the real world, interacted only with their invertebrate hosts. An entirely new and divergent view of some of these fungi was opened by the discovery that *B*. *bassiana* could occur as an endophyte in the tissues of maize plants and exerted a degree of natural biological control against European corn borer larvae, *Ostrinia nubilalis* (Lewis and Cossentine 1986; Bing and Lewis 1991). This endophytic “hidden” life for *Beauveria* that was so startling when first discovered is now known to be widespread and even rather common in a wide range of herbaceous and woody plants (e.g., Vidal and Jaber 2015; Barelli et al. 2016; Greenfield et al. 2016). More recently, it has been found that species of *Metarhizium* in particular can also lead a “secret” lives an endophyte (Behie et al. 2015; Barelli et al. 2016) but that these entomopathogenic fungi seem much more commonly to form natural rhizosphere associations with plant roots that are mutually beneficial (Bidochka et al. 2001; St. Leger 2008); these root–fungus–soil interactions seem to function in a manner that strongly resembles those involving ectomycorrhizal basidiomycetes. The physiological and genomic complexities of these cooperative interactions between plants and entomopathogenic fungi are now being studied actively (Pava-Ripoll et al. 2011; Behie and Bidochka 2014a, 2014b; Behie et al. 2015) and yielding some remarkable results.

## What secrets and surprises await discovery from the whole genomes of these fungi?

The full sequencing of any organismal genome establishes an enormous black box into which one may dip at random and pull out either something useful or something that is completely nonsensical unless that genome has been thoroughly and correctly annotated to point out what genes and other coding sequences are present and something about the roles of those annotated genes. What is often overlooked is that there may be much greater value in understanding how certain significant genes (e.g., mating type genes and those producing mycotoxins or affecting virulence for a host, etc.) are controlled (turned on or off, etc.), and what other parts of the genome may be essential to support the function of any target gene being studying. This annotation process is much slower and more exhaustive than the now increasingly rapid and inexpensive production and assembly of a full sequence. The potential importance of generating full genomic sequences from very large and very diverse range of organisms cannot be disputed and must not be underestimated. Nonetheless, I believe that it is more important to know what to do with an organismal genome, how to use that information to understand the overall function and biology of the organism, than it is to have generated the genome in the first place.

Even in some rather old literature (by contemporary molecular biological standards), there have been some intriguing findings for some cordycipitoid fungi that should be noted: It is well understood that the cell walls of true fungi (including all ascomycetes, of course) are chitinous, but studies with *Beauveria* and later also from *Metarhizium* (Pfeifer and Khachatourians 1987; Sandhu et al. 1999) indicated that protoplast release from these fungi was better if the protoplasting mixture incorporated cellulase. There appears to be no immediate explanation for such a phenomenon unless the presence of the cellulase somehow helps to support a better physical milieu for protoplast releases. Now that we know about the unexpected natural associations of *Beauveria* and *Metarhizium* with plants rather than with insects, as noted above, it would be potentially more important to explore the full genomic sequences of these and other cordycipitoid fungi to see if there are still unidentified sequences in them for any enzymes that are specifically linked to the biology of plants (e.g., any production of cellulose that might have been affected in these protoplasting mixes by the presence of the cellulase) or any enzymes that might be active in plants to help to establish or to perpetuate a stable plant–fungus association.

To ask about the presence of enzymes in presumably insect-pathogenic fungi that are active against plants or any components present in plants may seem unusual, but there is good reason to believe such unexpected capacities may exist more widely than has been appreciated. Again, knowing that fungi in general (with chitinous cell walls) and entomopathogenic fungi needing to be able to digest chitin to complete a successful infection process confirms what we already know: True fungi are able to generate chitinases that can have obvious utility to these organisms. On the other hand, however, Tribak et al. (2002) reported that *Isaria farinosa* can utilise microcrystalline cellulose as its sole source of carbon. This clearly points to the need to determine what other common and widely distributed insect-pathogenic fungi in the Hypocreales, at the very least, may have the ability to utilise plant-based substrates for growth and reproduction. How many of those fungi might prove to have the sort of alternative lifestyles that have been found now for the plant associations of *Beauveria* and *Metarhizium*? These are intriguing questions whose answers may become available by the careful screening of full genomic sequences for cordycipitoid fungi.

## Future prospects for working with cordycipitoid entomopathogenic fungi

The mating type genes, MAT1-1 and MAT1-2, for cordycipitoid and other perithecial ascomycetes are fairly well investigated (see the contribution from CordyForum 2016 by Chengshu Wang) for taxonomic purposes. Nonetheless, there are some critically important issues about the biology of these fungi intimately involve the mating type genes and their function but have received little active research attention: Despite the species of *Beauveria* and *Metarhizium* being the most widely distributed and common of all entomopathogenic fungi, why have the teleomorphic states of these fungi in *Cordyceps* and *Metarhizium* (formerly as *Metacordyceps* spp), respectively, been so very rarely discovered in nature? If the genomic and, subsequently, physical controls that determine when a sexual state will be formed were understood better, would those discoveries allow easier inductions of teleomorphic fruiting *in vitro* by such fungi as *O*. *sinensis*? Would it be possible to unlock or to understand the controls of the biochemical pathways that lead to the production of the pharmacologically invaluable compounds that have made *O. sinensis* and some other cordycipitoid fungi so economically important? Could the production of such compounds be modified or improved through directed modifications of the genome’s control sequences? Could the natural ecology of these fungi and their development be better understood? Of critical importance to these and all other questions that may be related to sexual reproduction in these fungi is *C*. *militaris* (as noted at the start of this discussion) since *C. militaris* is both the type species of *Cordyceps* and also the easiest of all cordycipitoid fungi to induce to produce its teleomorphs in culture. One can only hope that to understand how *C. militaris* operates at the genomic level would provide the means to unlock similar understandings in both laboratory and field settings for so many other cordycipitoid fungi.

The genetic and developmental manipulation of such fungi is not linked exclusively to the production of the teleomorphic stromata with their asci and ascospores. A great deal might be gained by acquiring a more genomically based understanding of two other essential sources of genetic diversity and gene flow in cordycipitoid fungi: Heterokaryosis (the presence of two or more genetically different nuclei in a common cytoplasm resulting from genetically controlled hyphal fusions) and parasexuality (so-called “mitotic recombination,” involving somatic diploidizations followed by rehaploidizations that might represent meioses and genetic reassortments occurring in an unexpected place and time in the life history, and without forming any teleomorphic structures) are both potent means by which temporary to permanent modifications of fungal genomes may occur. Both of these processes, if understood better, might allow natural breeding and selection to improve key properties of the fungi that differ significantly from the bioengineered, directed genomic modifications that also tend to raise problems for permits and licences from many national agencies that oversee biocontrol, biomedical, dietary, and other practical uses of these fungi.

Genomic studies will continue to lead and to expand the means to understand many of the developmental and biochemical pathways of the cordycipitoid fungi that make them so important for so many people globally but especially particularly in the age-old traditional herbal medicinal practices so widely used throughout China, Korea, Japan, and other eastern Asian regions. Will we finally be able to be more aware of the incompletely understood natural roles for the many mycotoxins that are known to be generated by entomopathogenic fungi? What new chemistries leading to complex biologically active compounds that might have enormous utility for medicine, agriculture, and public health await discovery from the genomes of these fungi? Might an understanding of genomes and how intracellular synthetic pathways in these fungi are controlled lead to greater and/or more readily predictable levels of production of compounds of interest? And, finally, as the already significant amount of scientifically sound pharmacological and medical research on the activities of compounds produced by cordycipitoid fungi increases and is better recognised globally, it will be very interesting to see if the market for these fungi and the products based on them will expand significantly beyond Asia.

It is clear that the large and wildly diverse universe of hypocrealean fungi pathogenic to insects, spiders, and other invertebrates revolves substantially around the potential uses of these fungi for the biological control of their hosts. Classically trained mycologists, entomologists, and invertebrate pathologists with deep interests in organismal biology feel some pain because of the lack of use of these fungi as biological control agents and would like to see a significant future expansion of such practical uses for them. To use fungi as practical biological control agents is a complex matter linking national and international laws and regulations, the needs of corporations, and the ability to produce, to formulate, and to apply infective fungal material (Humber 2016). Nonetheless, it is also clear from the presentations at Cordyceps Forum 2016 that there is a vast and extensive body of knowledge and active research focusing on completely different uses of these fungi. However, it is the more basic aspects of the research in this current period of genomically and molecularly driven studies on their taxonomy, systematics, biology, pharmaco-medical, and supplemental dietary uses of these fungi that formed the natural centre of interest for Cordyceps Forum 2016 and for the papers in this proceedings volume. These are exciting times for these fungi, and there are innumerable diverse directions in which knowledge can and must be expected to progress rapidly.
